# Intra- and inter­molecular proton transfer in 2,6-diamino­pyridinium 4-hy­droxy­pyridin-1-ium-2,6-dicarboxyl­ate

**DOI:** 10.1107/S1600536812037580

**Published:** 2012-09-05

**Authors:** Quoc-Cuong Ton, Michael Bolte

**Affiliations:** a Institut für Organische Chemie und Chemische Biologie, Goethe-Universität Frankfurt, Max-von-Laue-Strasse 7, 60438 Frankfurt am Main, Germany; b Institut für Anorganische und Analytische Chemie, Goethe-Universität Frankfurt, Max-von-Laue-Strasse 7, 60438 Frankfurt am Main, Germany

## Abstract

Chelidamic acid (4-hy­droxy­pyridine-2,6-dicarb­oxy­lic acid) and 2,6-diamino­pyridine react to form the title salt, C_5_H_8_N_3_^+^·C_7_H_4_NO_5_^−^; there are two formula units in the asymmetric unit. The pyridine N atom of 2,6-diamino­pyridine is protonated whereas chelidamic acid is deprotonated at both carboxyl­ate groups but protonated at the N atom; the reaction involves intra- and inter­molecular proton transfer. In the crystal, each 2,6-diamino­pyridinium cation participates in five strong N—H⋯O hydrogen bonds (including one bifurcated hydrogen bond). The crystal structure also features strong O—H⋯O hydrogen bonds between the chelidamate anions, leading to chains along the *a* axis.

## Related literature

For chelidamic acid, see: Tutughamiarso *et al.* (2012 ▶). For chelidamic acid monohydrate, see: Hall *et al.* (2000 ▶). For inter­action of chelidamic acid with heavy metal ions, see: Norkus *et al.* (2003 ▶). For supermolecular structures, see: Aakeröy *et al.* (2005 ▶); Brunsveld *et al.* (2001 ▶); Prins *et al.* (2001 ▶); Schmid & Mann (1954 ▶). For a description of the Cambridge Structural Database, see: Allen (2002 ▶).
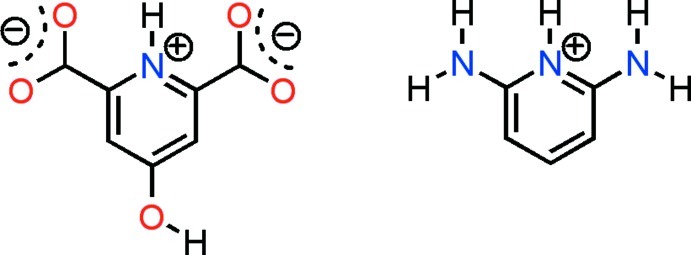


## Experimental

### 

#### Crystal data

C_5_H_8_N_3_^+^·C_7_H_4_NO_5_^−^
*M* *_r_* = 292.26
Orthorhombic, 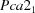

*a* = 14.963 (3) Å
*b* = 8.500 (2) Å
*c* = 20.385 (4) Å
*V* = 2592.7 (9) Å^3^
*Z* = 8
Mo *K*α radiation
μ = 0.12 mm^−1^
*T* = 173 K
0.40 × 0.30 × 0.20 mm

#### Data collection

Stoe IPDS II two-circle diffractometer
33672 measured reflections
2510 independent reflections
2243 reflections with *I* > 2σ(*I*)
*R*_int_ = 0.071

#### Refinement

*R*[*F* ^2^ > 2σ(*F* ^2^)] = 0.036
*wR*(*F* ^2^) = 0.083
*S* = 1.04
2510 reflections
417 parameters
1 restraint
H atoms treated by a mixture of independent and constrained refinement
Δρ_max_ = 0.16 e Å^−3^
Δρ_min_ = −0.23 e Å^−3^

### 

Data collection: *X-AREA* (Stoe & Cie, 2001 ▶); cell refinement: *X-AREA*; data reduction: *X-AREA*; program(s) used to solve structure: *SHELXS97* (Sheldrick, 2008 ▶); program(s) used to refine structure: *SHELXL97* (Sheldrick, 2008 ▶); molecular graphics: Mercury (Macrae *et al.*, 2008 ▶) and *XP* (Sheldrick, 2008 ▶); software used to prepare material for publication: *publCIF* (Westrip (2010 ▶).

## Supplementary Material

Crystal structure: contains datablock(s) I, global. DOI: 10.1107/S1600536812037580/ng5291sup1.cif

Structure factors: contains datablock(s) I. DOI: 10.1107/S1600536812037580/ng5291Isup2.hkl

Supplementary material file. DOI: 10.1107/S1600536812037580/ng5291Isup3.cml

Additional supplementary materials:  crystallographic information; 3D view; checkCIF report

## Figures and Tables

**Table 1:** Hydrogen-bond geometry (Å, °)

*D*—H⋯*A*	*D*—H	H⋯*A*	*D*⋯*A*	*D*—H⋯*A*
O5—H*O*5⋯O3^i^	0.84	1.67	2.495 (3)	169
O5′—H*O*5′⋯O3′^ii^	0.84	1.67	2.487 (3)	163
N21—H21*B*⋯O2^iii^	0.88 (5)	2.00 (5)	2.869 (4)	170 (4)
N22—H22*B*⋯O1′^iv^	0.86 (5)	2.04 (5)	2.892 (4)	169 (4)
N22′—H22*D*⋯O2′^v^	0.96 (4)	1.98 (4)	2.928 (4)	170 (4)
N1—H1*N*⋯O1	0.88 (3)	2.25 (3)	2.660 (3)	108 (3)
N1—H1*N*⋯O2	0.88 (3)	2.39 (4)	2.702 (4)	101 (2)
N1′—H1′*N*⋯O1′	0.87 (3)	2.32 (3)	2.669 (3)	104 (3)
N1′—H1′*N*⋯O2′	0.87 (3)	2.30 (4)	2.675 (4)	106 (2)
N11—H11*A*⋯O4	1.01 (4)	1.71 (3)	2.691 (4)	163 (4)
N11′—H11*B*⋯O4′	0.86 (3)	1.88 (3)	2.707 (4)	161 (4)
N21—H21*A*⋯O4	0.84 (5)	2.42 (5)	3.109 (4)	140 (5)
N21′—H21*C*⋯O2′	0.84 (5)	2.13 (6)	2.964 (4)	170 (5)
N21′—H21*D*⋯O1	0.90 (4)	2.03 (4)	2.916 (4)	169 (4)
N22—H22*A*⋯O2	1.03 (4)	2.01 (4)	3.040 (4)	177 (4)
N22′—H22*C*⋯O4′	0.94 (5)	2.40 (4)	3.130 (4)	134 (4)

Symmetry codes: (i) 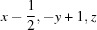; (ii) 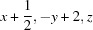; (iii) 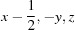; (iv) 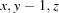; (v) 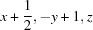.

**Fig. 1.**
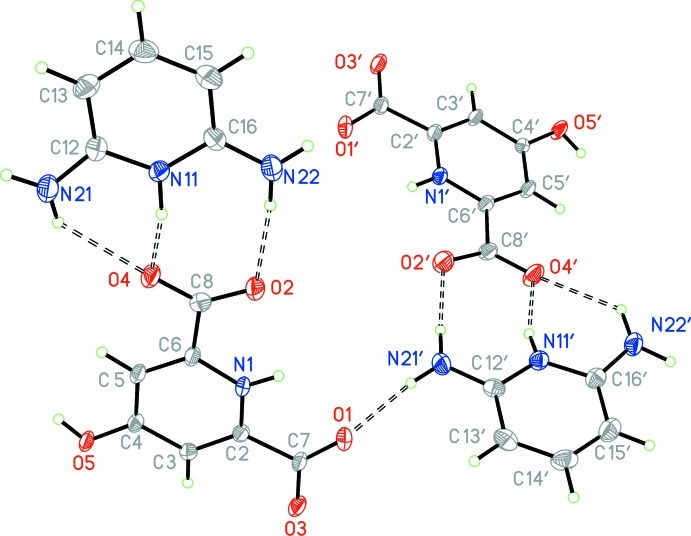
A perspective view of the title compound, showing the atom numbering scheme. Displacement ellipsoids are drawn at the 50% probability level and H atoms are shown as small spheres of arbitrary radii. Dashed lines indicate hydrogen bonds.

**Fig. 2.**
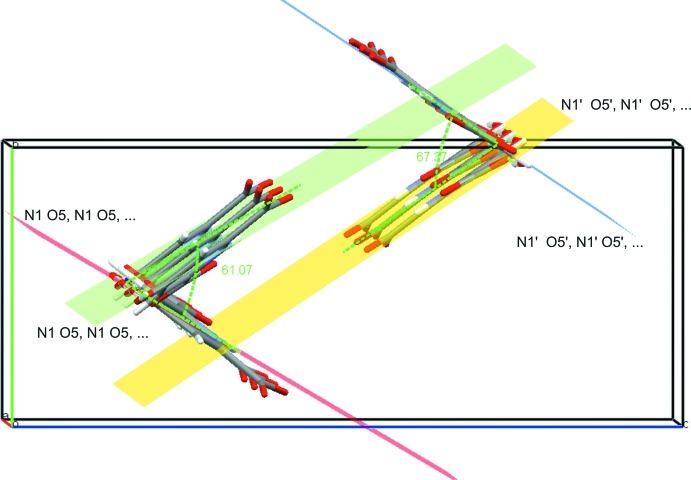
Four planes generated by chelidamates each gliding along the a-axis with at least four fragments. The planes are stretched over the defined N1 and O5 atoms (red or green) or N1' and O5' atoms (yellow or blue) whilst two planes are made up of symmetric chelidamates, which enclose angles of approximately 60°.

**Fig. 3.**
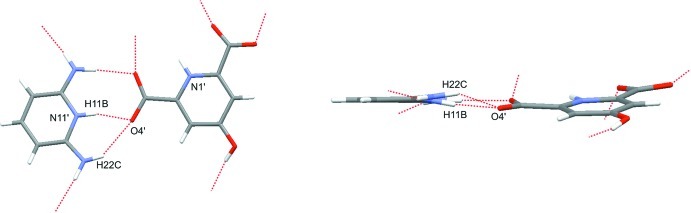
DDD–AA hydrogen bond interaction between atoms N1' and N11' of chelidamates and 2,6-diaminopyridininium (left). The out-of-plane drifted hydrogen atoms H11B and H22C; this effect is caused by a strong hydrogen-bond interaction with O4' (right). Red dashed lines indicate hydrogen bonds

**Fig. 4.**
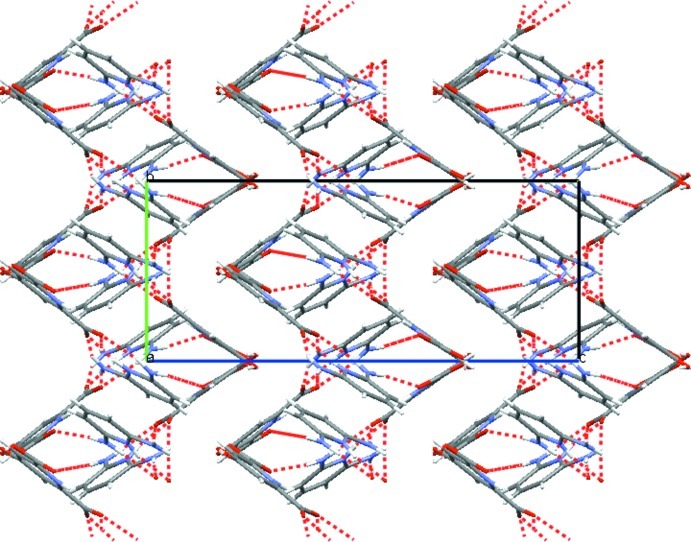
Crystal packing of the title compound viewed along the a-axis. Red dashed lines indicate the hydrogen-bond network which interlinks two chelidamates and two 2,6-diaminopyridinium cations to an entity that glides along the a-axis.

## supplementary crystallographic information

### Comment

The synthesis of new compounds by breaking and forming covalent bonds is conventional. A new challenge is creating materials consolidated by weak hydrogen bonds (Aakeröy *et al.*, 2005). The stability of these materials results mainly from the assembly of hydrogen-bonded networks within the structure or arises by cooperative effects as it has been exemplified in the manner of DNA or supramolecular polymers (Prins *et al.*, 2001; Brunsveld *et al.*, 2001). The object of this investigation was the co-crystallization of chelidamic acid I*a* and 2,6-diaminopyridine II in order to obtain the co-crystal III with an *ADA*/*DAD* pattern (*A*: hydrogen-bond acceptor, *D*: hydrogen-bond donor). However, the two components formed a salt (Fig. 1), which is reasonable considering the acid-base properties of the starting components. Chelidamic acid can exist in two tautomeric forms I*a* and I*b*. A search of the Cambridge Structural Database (CSD, Version 5.33 of November 2011, plus two updates; Allen, 2002) yielded no hits for chelidamic acid as neutral 4-hydroxypyridine or 4-pyridone tautomer. The structure of chelidamic acid monohydrate has been found to be zwitterionic [refcode KIXCUP (Hall *et al.*, 2000)] and two crystal structures involve chelidamic acid in coordination complexes (refcodes FEZHEY and FEZHIC). A recent study of three pseudopolymorphs of chelidamic acid has been carried out by Tutughamiarso *et al.* (2012). In general the different forms of chelidamic acid depend on the p*K_a_* values (Norkus *et al.*, 2003). In this study chelidamic acid is doubly deprotonated. The first proton transfer is assumed to be intramolecular (Hall *et al.*, 2000) while the second deprotonation is suggested to occur intermolecular with 2,6-diaminopyridine as proton acceptor. Compound II is known to be reactive in the presence of dicarboxylic acid anhydrides (Schmid & Mann, 1954) but a reaction with dicarboxylic acids without activation is not expected in this case. In the crystal structure of the title compound two one-dimensional hydrogen-bond networks are observed, connecting symmetry-equivalent chelidamates (generated by an *a* glide plane) *via* O—H···O chains, whilst those fragments are twisted approximately by 60° with respect to each other (Fig. 2). When comparing the symmetry-independent chelidamates with each other, a slight difference in planarity is noticeable. The plane stretching over all non-hydrogen atoms in the N1 or N1' unit shows a mean deviation of 0.053 Å and 0.078 Å [significant outliers: O3 = -0.109 (2), O4 = -0.121 (2) and O1' = -0.105 (2), O3' = 0.178 (2), O4' = 0.155 (2) Å], respectively. The H atom at O5 atom lies in the plane whereas the other H atom at O5' deviates by 0.125 (2) Å from it. In these two cases the aromatic ring atoms including the hydroxy groups were used for the definition of the plane [mean deviation from plane: 0.013 Å for O5 unit and 0.061 Å for O5' unit]. The planarity of the two symmetry-independent 2,6-diaminopyridinium cations is remarkable. A least-squares plane through all atoms of the N11 or N11' fragment yielded a mean deviation of 0.054 and 0.075 Å, respectively. The deviations of the H atoms H11B [0.19 (3) Å] and H22C [–0.17 (2) Å] from the mean plane are probably caused by bifurcated hydrogen bonds with the (also slightly out of plane drifted) O5' atom of chelidamate (Fig. 3). A further investigation of the crystal packing indicates a finite three-dimensional network between the four charged entities in the asymmetric unit, which run along the *a-*axis in a zigzag alignment (Fig. 4).

### Experimental

Chelidamic acid and 2,6-diaminopyridine are commercially available. Chelidamic acid was utilized without purification while 2,6-diaminopyridine had to be sublimed before use. A small amount of each compound was dissolved separately in approximately 15 drops of dimethyl sulfoxide (DMSO) before they were combined in a flask and set aside at room temperature. From the green-yellow mixture, block shaped crystals were obtained after several weeks.

### Refinement

Due to the absence of anomalous scatterers, 2351 Friedel pairs were merged. All H atoms were initially located by difference Fourier synthesis. Subsequently, H atoms bonded to C and O atoms were refined using a riding model, with C—H = 0.95 Å and O—H = 0.84 Å, and with *U*_iso_(H) = 1.2*U*_eq_(C) and 1.5*U*_eq_(O), respectively; H atoms bonded to O atoms were allowed to rotate about the C—O bond. H atoms bonded to N atoms were refined isotropically with *U*_iso_(H) = 1.2*U*_eq_(N).

### Crystal data

**Table:**

C_5_H_8_N_3_^+^·C_7_H_4_NO_5_^−^	*F*(000) = 1216
*M**_r_* = 292.26	*D*_x_ = 1.497 Mg m^−^^3^
Orthorhombic, *P**c**a*2_1_	Mo *K*α radiation, λ = 0.71073 Å
Hall symbol: P 2c -2ac	Cell parameters from 35668 reflections
*a* = 14.963 (3) Å	θ = 3.4–25.6°
*b* = 8.500 (2) Å	µ = 0.12 mm^−^^1^
*c* = 20.385 (4) Å	*T* = 173 K
*V* = 2592.7 (9) Å^3^	Block, colourless
*Z* = 8	0.40 × 0.30 × 0.20 mm

### Data collection

**Table:**

Stoe IPDS II two-circle diffractometer	2243 reflections with *I* > 2σ(*I*)
Radiation source: fine-focus sealed tube	*R*_int_ = 0.071
Graphite monochromator	θ_max_ = 25.6°, θ_min_ = 3.4°
ω scans	*h* = −18→18
33672 measured reflections	*k* = −10→10
2510 independent reflections	*l* = −24→24

### Refinement

**Table:**

Refinement on *F*^2^	Primary atom site location: structure-invariant direct methods
Least-squares matrix: full	Secondary atom site location: difference Fourier map
*R*[*F*^2^ > 2σ(*F*^2^)] = 0.036	Hydrogen site location: inferred from neighbouring sites
*wR*(*F*^2^) = 0.083	H atoms treated by a mixture of independent and constrained refinement
*S* = 1.04	*w* = 1/[σ^2^(*F*_o_^2^) + (0.0555*P*)^2^] where *P* = (*F*_o_^2^ + 2*F*_c_^2^)/3
2510 reflections	(Δ/σ)_max_ < 0.001
417 parameters	Δρ_max_ = 0.16 e Å^−^^3^
1 restraint	Δρ_min_ = −0.23 e Å^−^^3^

### Special details

**Table:**

Geometry. All e.s.d.'s (except the e.s.d. in the dihedral angle between two l.s. planes) are estimated using the full covariance matrix. The cell e.s.d.'s are taken into account individually in the estimation of e.s.d.'s in distances, angles and torsion angles; correlations between e.s.d.'s in cell parameters are only used when they are defined by crystal symmetry. An approximate (isotropic) treatment of cell e.s.d.'s is used for estimating e.s.d.'s involving l.s. planes.
Refinement. Refinement of *F*^2^ against ALL reflections. The weighted *R*-factor *wR* and goodness of fit *S* are based on *F*^2^, conventional *R*-factors *R* are based on *F*, with *F* set to zero for negative *F*^2^. The threshold expression of *F*^2^ > σ(*F*^2^) is used only for calculating *R*-factors(gt) *etc*. and is not relevant to the choice of reflections for refinement. *R*-factors based on *F*^2^ are statistically about twice as large as those based on *F*, and *R*- factors based on ALL data will be even larger.

### Fractional atomic coordinates and isotropic or equivalent isotropic displacement parameters (Å^2^)

**Table:**

	*x*	*y*	*z*	*U*_iso_*/*U*_eq_	
N1	0.28490 (16)	0.3325 (3)	0.29385 (13)	0.0189 (5)	
H1N	0.327 (2)	0.296 (4)	0.3201 (19)	0.023*	
O1	0.45940 (13)	0.3882 (3)	0.28520 (12)	0.0274 (5)	
O2	0.23936 (14)	0.1442 (3)	0.39526 (12)	0.0290 (5)	
O3	0.44259 (14)	0.5304 (3)	0.19256 (12)	0.0247 (5)	
O4	0.09524 (13)	0.1412 (3)	0.36286 (12)	0.0284 (5)	
O5	0.10342 (13)	0.5135 (3)	0.16602 (12)	0.0218 (5)	
HO5	0.0514	0.4868	0.1767	0.033*	
C2	0.31328 (18)	0.4270 (3)	0.24492 (15)	0.0179 (6)	
C3	0.2527 (2)	0.4901 (4)	0.20125 (18)	0.0188 (7)	
H3	0.2726	0.5567	0.1668	0.023*	
C4	0.16070 (18)	0.4548 (4)	0.20811 (16)	0.0180 (6)	
C5	0.13420 (18)	0.3556 (4)	0.26059 (15)	0.0179 (6)	
H5	0.0728	0.3311	0.2667	0.021*	
C6	0.19726 (18)	0.2947 (4)	0.30257 (14)	0.0180 (6)	
C7	0.41412 (18)	0.4510 (4)	0.24134 (15)	0.0199 (6)	
C8	0.17574 (19)	0.1826 (4)	0.35877 (15)	0.0216 (7)	
N11	0.05045 (16)	−0.0540 (3)	0.46143 (14)	0.0211 (6)	
H11A	0.077 (3)	0.005 (4)	0.426 (2)	0.025*	
N21	−0.0790 (2)	−0.0368 (4)	0.40028 (18)	0.0333 (7)	
H21A	−0.051 (3)	0.024 (6)	0.375 (3)	0.040*	
H21B	−0.136 (3)	−0.060 (5)	0.395 (2)	0.040*	
N22	0.18961 (18)	−0.0644 (4)	0.51078 (16)	0.0298 (7)	
H22A	0.209 (3)	0.006 (5)	0.472 (2)	0.036*	
H22B	0.222 (3)	−0.080 (5)	0.545 (2)	0.036*	
C12	−0.0384 (2)	−0.0884 (4)	0.45527 (17)	0.0229 (7)	
C13	−0.0804 (2)	−0.1742 (4)	0.50451 (18)	0.0297 (8)	
H13	−0.1427	−0.1958	0.5027	0.036*	
C14	−0.0283 (2)	−0.2278 (4)	0.55677 (17)	0.0310 (8)	
H14	−0.0561	−0.2872	0.5906	0.037*	
C15	0.0634 (2)	−0.1973 (4)	0.56117 (16)	0.0283 (7)	
H15	0.0979	−0.2375	0.5965	0.034*	
C16	0.1027 (2)	−0.1060 (4)	0.51205 (16)	0.0226 (7)	
N1'	0.45633 (16)	0.8257 (3)	0.62319 (13)	0.0193 (5)	
H1'N	0.420 (2)	0.782 (4)	0.5953 (19)	0.023*	
O1'	0.27918 (14)	0.8557 (3)	0.63201 (12)	0.0289 (5)	
O2'	0.50769 (14)	0.6478 (3)	0.52175 (12)	0.0322 (6)	
O3'	0.29024 (14)	0.9805 (3)	0.72949 (13)	0.0273 (6)	
O4'	0.65252 (14)	0.6636 (3)	0.55187 (13)	0.0323 (6)	
O5'	0.62710 (14)	1.0323 (3)	0.75228 (13)	0.0272 (6)	
HO5'	0.6794	1.0350	0.7373	0.041*	
C2'	0.42327 (18)	0.9094 (4)	0.67481 (15)	0.0190 (6)	
C3'	0.47987 (19)	0.9782 (4)	0.71856 (18)	0.0195 (7)	
H3'	0.4569	1.0356	0.7548	0.023*	
C4'	0.57377 (18)	0.9632 (4)	0.70940 (15)	0.0180 (6)	
C5'	0.60514 (18)	0.8727 (4)	0.65626 (16)	0.0208 (7)	
H5'	0.6675	0.8597	0.6494	0.025*	
C6'	0.54480 (19)	0.8037 (4)	0.61458 (15)	0.0188 (6)	
C7'	0.32115 (18)	0.9150 (4)	0.67830 (16)	0.0193 (6)	
C8'	0.57095 (19)	0.6954 (4)	0.55701 (16)	0.0222 (7)	
N11'	0.69417 (17)	0.4557 (3)	0.45602 (14)	0.0216 (6)	
H11B	0.670 (3)	0.530 (5)	0.482 (2)	0.026*	
N21'	0.55525 (19)	0.4508 (4)	0.40698 (16)	0.0290 (6)	
H21C	0.538 (3)	0.514 (5)	0.436 (3)	0.035*	
H21D	0.520 (3)	0.426 (5)	0.373 (2)	0.035*	
N22'	0.82341 (19)	0.4676 (4)	0.51780 (17)	0.0304 (7)	
H22C	0.788 (3)	0.512 (5)	0.551 (3)	0.036*	
H22D	0.886 (3)	0.442 (5)	0.517 (2)	0.036*	
C12'	0.6415 (2)	0.4059 (4)	0.40541 (16)	0.0221 (6)	
C13'	0.6800 (2)	0.3114 (4)	0.35694 (16)	0.0279 (7)	
H13'	0.6452	0.2723	0.3215	0.034*	
C14'	0.7703 (2)	0.2761 (4)	0.36190 (18)	0.0314 (8)	
H14'	0.7971	0.2140	0.3285	0.038*	
C15'	0.8230 (2)	0.3275 (4)	0.41358 (18)	0.0298 (8)	
H15'	0.8849	0.3027	0.4154	0.036*	
C16'	0.7828 (2)	0.4164 (4)	0.46261 (16)	0.0225 (7)	

### Atomic displacement parameters (Å^2^)

**Table:**

	*U*^11^	*U*^22^	*U*^33^	*U*^12^	*U*^13^	*U*^23^
N1	0.0105 (11)	0.0253 (14)	0.0208 (13)	−0.0001 (10)	−0.0017 (10)	0.0004 (11)
O1	0.0146 (10)	0.0403 (14)	0.0275 (12)	−0.0003 (9)	−0.0053 (9)	0.0016 (10)
O2	0.0183 (10)	0.0420 (15)	0.0267 (12)	0.0010 (9)	−0.0020 (9)	0.0121 (11)
O3	0.0119 (10)	0.0352 (13)	0.0269 (13)	−0.0014 (8)	0.0029 (8)	0.0075 (10)
O4	0.0162 (10)	0.0398 (14)	0.0292 (12)	−0.0061 (9)	−0.0008 (9)	0.0118 (11)
O5	0.0100 (10)	0.0319 (13)	0.0233 (13)	0.0007 (8)	−0.0011 (9)	0.0082 (9)
C2	0.0142 (13)	0.0207 (15)	0.0187 (14)	−0.0009 (10)	0.0006 (11)	−0.0032 (12)
C3	0.0167 (15)	0.0236 (17)	0.0162 (17)	−0.0003 (12)	0.0007 (12)	0.0026 (12)
C4	0.0130 (14)	0.0209 (16)	0.0202 (16)	0.0043 (11)	0.0004 (11)	−0.0016 (13)
C5	0.0106 (12)	0.0206 (15)	0.0225 (15)	−0.0015 (10)	0.0003 (11)	−0.0018 (12)
C6	0.0114 (13)	0.0218 (16)	0.0208 (15)	0.0005 (11)	−0.0010 (11)	−0.0010 (12)
C7	0.0106 (12)	0.0265 (16)	0.0224 (15)	0.0010 (11)	−0.0006 (11)	−0.0038 (13)
C8	0.0201 (15)	0.0253 (17)	0.0195 (16)	0.0008 (12)	0.0025 (12)	−0.0001 (13)
N11	0.0178 (12)	0.0252 (15)	0.0203 (15)	−0.0013 (10)	0.0005 (10)	0.0053 (12)
N21	0.0213 (14)	0.0440 (19)	0.0345 (18)	−0.0053 (14)	−0.0044 (12)	0.0115 (17)
N22	0.0223 (14)	0.0395 (19)	0.0276 (17)	−0.0028 (12)	−0.0058 (12)	0.0064 (15)
C12	0.0200 (14)	0.0222 (17)	0.0264 (16)	−0.0006 (12)	−0.0004 (12)	−0.0014 (14)
C13	0.0250 (15)	0.0327 (19)	0.0315 (19)	−0.0076 (13)	0.0048 (13)	0.0032 (16)
C14	0.0383 (18)	0.031 (2)	0.0241 (18)	−0.0091 (15)	0.0017 (15)	0.0039 (15)
C15	0.0333 (17)	0.0291 (19)	0.0226 (18)	−0.0006 (14)	−0.0017 (14)	0.0035 (14)
C16	0.0253 (15)	0.0232 (16)	0.0193 (16)	0.0026 (13)	−0.0032 (12)	0.0000 (14)
N1'	0.0141 (11)	0.0254 (14)	0.0183 (13)	−0.0019 (10)	−0.0012 (10)	−0.0032 (11)
O1'	0.0160 (9)	0.0417 (15)	0.0289 (13)	0.0004 (9)	−0.0073 (9)	−0.0017 (11)
O2'	0.0238 (11)	0.0443 (15)	0.0286 (13)	−0.0032 (10)	0.0007 (10)	−0.0118 (11)
O3'	0.0122 (10)	0.0409 (14)	0.0288 (14)	0.0006 (9)	0.0015 (9)	−0.0072 (11)
O4'	0.0194 (10)	0.0435 (15)	0.0341 (13)	0.0019 (10)	0.0035 (10)	−0.0177 (12)
O5'	0.0110 (9)	0.0443 (14)	0.0263 (14)	−0.0065 (9)	0.0008 (9)	−0.0127 (11)
C2'	0.0137 (13)	0.0208 (15)	0.0225 (15)	0.0011 (11)	0.0015 (12)	0.0036 (13)
C3'	0.0112 (13)	0.0237 (16)	0.0237 (19)	0.0010 (11)	0.0025 (12)	−0.0017 (13)
C4'	0.0132 (14)	0.0249 (16)	0.0159 (16)	−0.0005 (12)	0.0008 (11)	−0.0007 (13)
C5'	0.0121 (14)	0.0257 (16)	0.0247 (16)	0.0008 (12)	0.0032 (11)	−0.0001 (13)
C6'	0.0142 (13)	0.0207 (16)	0.0215 (16)	0.0002 (11)	0.0039 (11)	0.0018 (12)
C7'	0.0138 (13)	0.0216 (15)	0.0225 (15)	−0.0005 (11)	−0.0011 (12)	0.0037 (13)
C8'	0.0185 (14)	0.0269 (17)	0.0213 (16)	−0.0038 (12)	0.0019 (12)	−0.0049 (13)
N11'	0.0196 (13)	0.0227 (15)	0.0225 (15)	0.0015 (11)	0.0006 (10)	−0.0039 (13)
N21'	0.0238 (14)	0.0394 (18)	0.0239 (16)	0.0041 (12)	−0.0062 (12)	−0.0083 (14)
N22'	0.0180 (14)	0.0435 (18)	0.0297 (18)	0.0012 (13)	−0.0043 (12)	−0.0067 (16)
C12'	0.0264 (15)	0.0209 (16)	0.0190 (16)	−0.0027 (12)	0.0018 (12)	0.0029 (14)
C13'	0.0363 (17)	0.0253 (18)	0.0222 (17)	−0.0002 (14)	−0.0042 (13)	−0.0022 (14)
C14'	0.0385 (19)	0.0288 (19)	0.0269 (18)	0.0083 (14)	0.0077 (14)	−0.0056 (15)
C15'	0.0248 (15)	0.0298 (19)	0.035 (2)	0.0060 (13)	0.0028 (14)	−0.0004 (15)
C16'	0.0214 (14)	0.0202 (16)	0.0258 (17)	−0.0016 (11)	0.0026 (12)	0.0019 (14)

### Geometric parameters (Å, º)

**Table:**

N1—C2	1.349 (4)	N1'—C6'	1.348 (4)
N1—C6	1.362 (4)	N1'—C2'	1.363 (4)
N1—H1N	0.88 (4)	N1'—H1'N	0.87 (4)
O1—C7	1.242 (4)	O1'—C7'	1.241 (4)
O2—C8	1.251 (4)	O2'—C8'	1.256 (4)
O3—C7	1.275 (4)	O3'—C7'	1.270 (4)
O4—C8	1.258 (4)	O4'—C8'	1.254 (4)
O5—C4	1.311 (4)	O5'—C4'	1.322 (4)
O5—HO5	0.8400	O5'—HO5'	0.8400
C2—C3	1.379 (4)	C2'—C3'	1.362 (5)
C2—C7	1.524 (4)	C2'—C7'	1.530 (4)
C3—C4	1.416 (4)	C3'—C4'	1.423 (4)
C3—H3	0.9500	C3'—H3'	0.9500
C4—C5	1.419 (5)	C4'—C5'	1.409 (4)
C5—C6	1.375 (4)	C5'—C6'	1.372 (4)
C5—H5	0.9500	C5'—H5'	0.9500
C6—C8	1.524 (4)	C6'—C8'	1.542 (4)
N11—C12	1.367 (4)	N11'—C12'	1.365 (4)
N11—C16	1.368 (4)	N11'—C16'	1.374 (4)
N11—H11A	0.97 (4)	N11'—H11B	0.90 (4)
N21—C12	1.349 (5)	N21'—C12'	1.346 (4)
N21—H21A	0.84 (5)	N21'—H21C	0.84 (5)
N21—H21B	0.88 (5)	N21'—H21D	0.89 (5)
N22—C16	1.349 (4)	N22'—C16'	1.351 (5)
N22—H22A	1.04 (5)	N22'—H22C	0.94 (5)
N22—H22B	0.86 (5)	N22'—H22D	0.96 (4)
C12—C13	1.391 (5)	C12'—C13'	1.398 (5)
C13—C14	1.397 (5)	C13'—C14'	1.388 (5)
C13—H13	0.9500	C13'—H13'	0.9500
C14—C15	1.399 (5)	C14'—C15'	1.387 (5)
C14—H14	0.9500	C14'—H14'	0.9500
C15—C16	1.396 (5)	C15'—C16'	1.390 (5)
C15—H15	0.9500	C15'—H15'	0.9500
			
C2—N1—C6	122.7 (3)	C6'—N1'—C2'	121.9 (3)
C2—N1—H1N	116 (2)	C6'—N1'—H1'N	118 (2)
C6—N1—H1N	121 (2)	C2'—N1'—H1'N	120 (2)
C4—O5—HO5	109.5	C4'—O5'—HO5'	109.5
N1—C2—C3	120.1 (3)	C3'—C2'—N1'	120.3 (3)
N1—C2—C7	115.2 (3)	C3'—C2'—C7'	125.3 (3)
C3—C2—C7	124.6 (3)	N1'—C2'—C7'	114.5 (3)
C2—C3—C4	119.5 (3)	C2'—C3'—C4'	119.3 (3)
C2—C3—H3	120.2	C2'—C3'—H3'	120.3
C4—C3—H3	120.2	C4'—C3'—H3'	120.3
O5—C4—C3	119.4 (3)	O5'—C4'—C5'	123.4 (3)
O5—C4—C5	122.5 (3)	O5'—C4'—C3'	118.0 (3)
C3—C4—C5	118.2 (3)	C5'—C4'—C3'	118.6 (3)
C6—C5—C4	120.1 (2)	C6'—C5'—C4'	119.4 (3)
C6—C5—H5	120.0	C6'—C5'—H5'	120.3
C4—C5—H5	120.0	C4'—C5'—H5'	120.3
N1—C6—C5	119.4 (3)	N1'—C6'—C5'	120.4 (3)
N1—C6—C8	116.7 (2)	N1'—C6'—C8'	115.5 (3)
C5—C6—C8	123.9 (3)	C5'—C6'—C8'	124.0 (3)
O1—C7—O3	127.3 (3)	O1'—C7'—O3'	128.2 (3)
O1—C7—C2	116.6 (3)	O1'—C7'—C2'	117.2 (3)
O3—C7—C2	116.0 (3)	O3'—C7'—C2'	114.5 (3)
O2—C8—O4	128.1 (3)	O4'—C8'—O2'	128.0 (3)
O2—C8—C6	116.7 (3)	O4'—C8'—C6'	116.0 (3)
O4—C8—C6	115.3 (3)	O2'—C8'—C6'	115.9 (3)
C12—N11—C16	123.8 (3)	C12'—N11'—C16'	123.7 (3)
C12—N11—H11A	116 (2)	C12'—N11'—H11B	116 (3)
C16—N11—H11A	120 (2)	C16'—N11'—H11B	120 (3)
C12—N21—H21A	119 (3)	C12'—N21'—H21C	120 (3)
C12—N21—H21B	117 (3)	C12'—N21'—H21D	118 (3)
H21A—N21—H21B	123 (5)	H21C—N21'—H21D	121 (4)
C16—N22—H22A	116 (2)	C16'—N22'—H22C	118 (3)
C16—N22—H22B	119 (3)	C16'—N22'—H22D	110 (3)
H22A—N22—H22B	123 (4)	H22C—N22'—H22D	131 (4)
N21—C12—N11	116.4 (3)	N21'—C12'—N11'	116.6 (3)
N21—C12—C13	124.6 (3)	N21'—C12'—C13'	125.0 (3)
N11—C12—C13	119.0 (3)	N11'—C12'—C13'	118.4 (3)
C12—C13—C14	118.0 (3)	C14'—C13'—C12'	118.2 (3)
C12—C13—H13	121.0	C14'—C13'—H13'	120.9
C14—C13—H13	121.0	C12'—C13'—H13'	120.9
C13—C14—C15	122.4 (3)	C15'—C14'—C13'	122.8 (3)
C13—C14—H14	118.8	C15'—C14'—H14'	118.6
C15—C14—H14	118.8	C13'—C14'—H14'	118.6
C16—C15—C14	118.0 (3)	C14'—C15'—C16'	118.1 (3)
C16—C15—H15	121.0	C14'—C15'—H15'	121.0
C14—C15—H15	121.0	C16'—C15'—H15'	121.0
N22—C16—N11	116.9 (3)	N22'—C16'—N11'	115.9 (3)
N22—C16—C15	124.4 (3)	N22'—C16'—C15'	125.4 (3)
N11—C16—C15	118.7 (3)	N11'—C16'—C15'	118.7 (3)
			
C6—N1—C2—C3	0.2 (4)	C6'—N1'—C2'—C3'	2.1 (5)
C6—N1—C2—C7	−177.7 (3)	C6'—N1'—C2'—C7'	−177.1 (3)
N1—C2—C3—C4	−0.1 (5)	N1'—C2'—C3'—C4'	0.7 (5)
C7—C2—C3—C4	177.7 (3)	C7'—C2'—C3'—C4'	179.8 (3)
C2—C3—C4—O5	−179.2 (3)	C2'—C3'—C4'—O5'	179.4 (3)
C2—C3—C4—C5	0.4 (5)	C2'—C3'—C4'—C5'	−2.1 (5)
O5—C4—C5—C6	178.7 (3)	O5'—C4'—C5'—C6'	179.3 (3)
C3—C4—C5—C6	−0.9 (5)	C3'—C4'—C5'—C6'	0.8 (5)
C2—N1—C6—C5	−0.7 (4)	C2'—N1'—C6'—C5'	−3.4 (5)
C2—N1—C6—C8	178.2 (3)	C2'—N1'—C6'—C8'	175.3 (3)
C4—C5—C6—N1	1.0 (4)	C4'—C5'—C6'—N1'	1.9 (5)
C4—C5—C6—C8	−177.8 (3)	C4'—C5'—C6'—C8'	−176.7 (3)
N1—C2—C7—O1	−3.3 (4)	C3'—C2'—C7'—O1'	175.0 (3)
C3—C2—C7—O1	178.8 (3)	N1'—C2'—C7'—O1'	−5.8 (4)
N1—C2—C7—O3	175.0 (3)	C3'—C2'—C7'—O3'	−5.3 (4)
C3—C2—C7—O3	−2.8 (4)	N1'—C2'—C7'—O3'	173.9 (3)
N1—C6—C8—O2	5.3 (4)	N1'—C6'—C8'—O4'	−174.3 (3)
C5—C6—C8—O2	−175.9 (3)	C5'—C6'—C8'—O4'	4.4 (5)
N1—C6—C8—O4	−175.3 (3)	N1'—C6'—C8'—O2'	4.5 (4)
C5—C6—C8—O4	3.5 (5)	C5'—C6'—C8'—O2'	−176.8 (3)
C16—N11—C12—N21	−175.8 (3)	C16'—N11'—C12'—N21'	178.1 (3)
C16—N11—C12—C13	3.5 (5)	C16'—N11'—C12'—C13'	−0.9 (5)
N21—C12—C13—C14	176.2 (4)	N21'—C12'—C13'—C14'	179.6 (3)
N11—C12—C13—C14	−3.0 (5)	N11'—C12'—C13'—C14'	−1.5 (5)
C12—C13—C14—C15	0.4 (5)	C12'—C13'—C14'—C15'	1.6 (5)
C13—C14—C15—C16	1.8 (5)	C13'—C14'—C15'—C16'	0.9 (5)
C12—N11—C16—N22	178.9 (3)	C12'—N11'—C16'—N22'	−175.9 (3)
C12—N11—C16—C15	−1.2 (5)	C12'—N11'—C16'—C15'	3.4 (5)
C14—C15—C16—N22	178.4 (3)	C14'—C15'—C16'—N22'	176.0 (3)
C14—C15—C16—N11	−1.4 (5)	C14'—C15'—C16'—N11'	−3.2 (5)

### Hydrogen-bond geometry (Å, º)

**Table:**

*D*—H···*A*	*D*—H	H···*A*	*D*···*A*	*D*—H···*A*
O5—H*O*5···O3^i^	0.84	1.67	2.495 (3)	169
O5′—H*O*5′···O3′^ii^	0.84	1.67	2.487 (3)	163
N21—H21*B*···O2^iii^	0.88 (5)	2.00 (5)	2.869 (4)	170 (4)
N22—H22*B*···O1′^iv^	0.86 (5)	2.04 (5)	2.892 (4)	169 (4)
N22′—H22*D*···O2′^v^	0.96 (4)	1.98 (4)	2.928 (4)	170 (4)
N1—H1*N*···O1	0.88 (3)	2.25 (3)	2.660 (3)	108 (3)
N1—H1*N*···O2	0.88 (3)	2.39 (4)	2.702 (4)	101 (2)
N1′—H1′*N*···O1′	0.87 (3)	2.32 (3)	2.669 (3)	104 (3)
N1′—H1′*N*···O2′	0.87 (3)	2.30 (4)	2.675 (4)	106 (2)
N11—H11*A*···O4	1.01 (4)	1.71 (3)	2.691 (4)	163 (4)
N11′—H11*B*···O4′	0.86 (3)	1.88 (3)	2.707 (4)	161 (4)
N21—H21*A*···O4	0.84 (5)	2.42 (5)	3.109 (4)	140 (5)
N21′—H21*C*···O2′	0.84 (5)	2.13 (6)	2.964 (4)	170 (5)
N21′—H21*D*···O1	0.90 (4)	2.03 (4)	2.916 (4)	169 (4)
N22—H22*A*···O2	1.03 (4)	2.01 (4)	3.040 (4)	177 (4)
N22′—H22*C*···O4′	0.94 (5)	2.40 (4)	3.130 (4)	134 (4)

Symmetry codes: (i) *x*−1/2, −*y*+1, *z*; (ii) *x*+1/2, −*y*+2, *z*; (iii) *x*−1/2, −*y*, *z*; (iv) *x*, *y*−1, *z*; (v) *x*+1/2, −*y*+1, *z*.

## References

[bb1] Aakeröy, B. C., Desper, J. & Urbina, F. J. (2005). *Chem. Commun.* pp. 2820–2822. 10.1039/b503718b 15928769

[bb12] Allen, F. H. (2002). *Acta Cryst.* B**58**, 380–388. 10.1107/s0108768102003890 12037359

[bb2] Brunsveld, L., Folmer, B. J. B., Meijer, E. W. & Sijbesma, R. P. (2001). *Chem. Rev.* **101**, 4071–4097. 10.1021/cr990125q 11740927

[bb3] Hall, A. K., Harrowfield, J. M., Skelton, B. W. & White, A. H. (2000). *Acta Cryst.* C**56**, 448–450. 10.1107/S0108270199015620 10815204

[bb4] Macrae, C. F., Bruno, I. J., Chisholm, J. A., Edgington, P. R., McCabe, P., Pidcock, E., Rodriguez-Monge, L., Taylor, R., van de Streek, J. & Wood, P. A. (2008). *J* *Appl* *Cryst* **41**, 466–470.

[bb5] Norkus, E., Stalnioniene, I. & Crans, D. C. (2003). *Heteroat* *Chem.* **14**, 625–632.

[bb6] Prins, L. J., Reinhoudt, D. N. & Timmerman, P. (2001). *Angew. Chem. Int. Ed. Engl.* **40**, 2382–2426. 10.1002/1521-3773(20010702)40:13<2382::aid-anie2382>3.0.co;2-g 11443654

[bb7] Schmid, L. & Mann, H. (1954). *Chem. Monthly*, **85**, 864–871.

[bb8] Sheldrick, G. M. (2008). *Acta Cryst.* A**64**, 112–122. 10.1107/S0108767307043930 18156677

[bb9] Stoe & Cie (2001). *X-AREA* Stoe & Cie, Darmstadt, Germany.

[bb10] Tutughamiarso, M., Pisternick, T. & Egert, E. (2012). *Acta Cryst.* C**68**, o344–o350. 10.1107/S0108270112031691 22935501

[bb11] Westrip, S. P. (2010). *J. Appl. Cryst.* **43**, 920–925.

